# Calcium and Vitamin D in the Regulation of Energy Balance: Where Do We Stand?

**DOI:** 10.3390/ijms15034938

**Published:** 2014-03-20

**Authors:** Mario J. Soares, Kaveri Pathak, Emily K. Calton

**Affiliations:** Directorate of Nutrition, Dietetics & Food Science, School of Public Health, Faculty of Health Sciences, Curtin University, Perth 6845, Australia; E-Mails: kaveri.pathak@postgrad.curtin.edu.au (K.P.); emily.calton@curtin.edu.au (E.K.C.)

**Keywords:** calcium, vitamin D, obesity, energy balance, chronic disease

## Abstract

There is a pandemic of obesity and associated chronic diseases. Dietary calcium and vitamin D have many extra-skeletal roles in human health. In this review we have summarized the current understanding of their influence on human energy balance by examining the epidemiological, clinical, animal, cellular and molecular evidence. We opine that while calcium and vitamin D are functional nutrients in the battle against obesity, there is a need for prospective human trials to tilt the balance of evidence in favour of these nutrients.

## 1. Introduction

Prevalence estimates of obesity are well over 50% in most WHO regions of the world, except South-East Asia. The observations that poor calcium intakes and inadequate vitamin D status are associated with chronic diseases like obesity, cardiovascular disease and type 2 diabetes (T2DM), hence become increasingly important [1–3]. Such relationships between nutrients and disease offer a potential public health strategy, if concerned agencies accept the available balance of evidence. Calcium and vitamin D have many biological effects and determining their requirements for health would depend on which endpoint is of greatest concern. Hence nutrient intake or nutrient status adequacy as judged against one biological endpoint may not be appropriate for other putative functions. Central to these issues is the precise cut off used to determine adequacy of nutrient status. In the case of vitamin D, while it is accepted that a value <25 nmol/L denotes deficiency, there is still disagreement whether >50 nmol/L signifies adequacy of function. There is the viewpoint that even for skeletal effects a value >75 nmol/L should be the norm [3–9]. Moreover given the dramatic increase in techniques for the determination of 25(OH)D standardisation of results between methods and laboratories is a problem [10,11]. So after a thorough review of the evidence available, a 2010 report by the Institute of Medicine advocated calcium and vitamin D only for bone health [12]. In this paper we summarize the evidence on their extra-skeletal benefits as they pertain to energy balance and obesity.

## 2. Calcium and Body Weight

**Figure 1.**
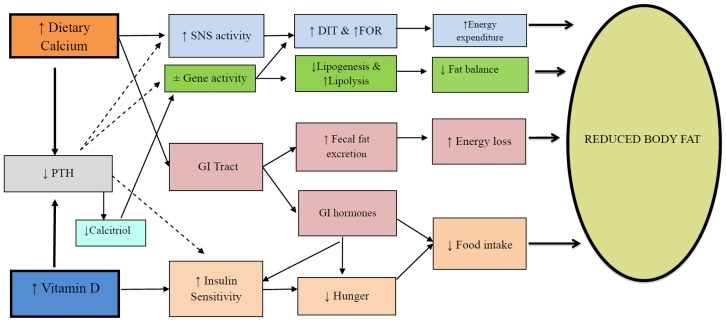
The modulation of energy balance by calcium and vitamin D. ↑ = increased, ↓ = decreased, PTH = parathyroid hormone, Calcitriol = 1,25(OH_2_)D, SNS = sympathetic nervous system, ±Gene activity = decreased expression of genes controlling lipogenesis (usually sterol regulatory element-binding protein-1, peroxisome proliferator-activated receptor gamma, fatty acid synthase) or increased expression of genes for lipolysis (lipoprotein lipase or hormone sensitive lipase) and increased thermogenesis (uncoupling protein activity), GI = gastro intestinal, DIT = diet-induced thermogenesis, FOR = fat oxidation rate. (From Soares *et al.* 2012 [8] with permission).

An inverse relationship between calcium intake and body weight was first noted by McCarron *et al.* [13]. However interest in the area was sparked by observation that increasing the intake of calcium through yoghurt increased the loss of body fat particularly from the abdominal region [14]. Based primarily on studies with the agouti mouse model, Zemel *et al*. [14] proposed that intracellular calcium (iCa^2+^) was key to fat deposition and hence obesity. According to this early scheme increases in dietary calcium would, via PTH, chronically lower iCa^2+^ in the adipocyte. This would then act to reciprocally reduce lipid deposition while stimulating adipose tissue breakdown. We have updated this model to include several other potential pathways (Figure 1) that could influence both sides of the energy balance equation in humans [15,16]. A review of available randomised controlled trials (RCTs) led us to conclude that, ingested calcium could have two effects [16]: (1) An increase in whole body oxidation of fat. Carbohydrate and protein balances are readily achieved over the short term as fluctuations in intake are offset by reciprocal changes in substrate oxidation. In contrast an increase in fat oxidation will lag an increase in fat intake, leading to fat storage. It follows that any nutrient which acutely or chronically raises whole body fat oxidation would result in a less positive fat balance, all other things being equal. A meta-analysis by an independent group has confirmed that increasing calcium by ~800 mg/day would favour an 11% increase in fat oxidation [17]; (2) An increase in faecal fat excretion. Within the gastro intestinal tract unabsorbed calcium links with dietary fat to form insoluble calcium-fatty acid soaps that are excreted. This presents a pathway of faecal energy loss and a predisposition to a negative energy balance. Available data indicates for every ~1200 mg/day of calcium, one can predict an excretion of ~5 g/day of fat or 45 kcal/day [18]. Future studies based on dose response trials could determine the optimum intake for these two actions of calcium.

A greater fat oxidation and increased faecal fat excretion through increased calcium intake will not result in an instant shedding of all excess body fat. So far reviews of RCTs show either no or a small augmentation of weight and fat loss (~0.7 to 0.9 kg) from calcium whether with or without vitamin D [19,20]. This will change as more prospective trials demonstrate their beneficial effect [21,22]. Interestingly, there is also evidence that despite no change in total fat, calcium may also increase the loss of visceral adipose tissue [21,23]. Overall, while such small decreases (<1 kg) in fat mass would be treated as clinically non-significant, they could have a dramatic effect on secular weight gain at the population level [24].

## 3. Vitamin D and Energy Regulation

Animal and cellular studies strongly indicate a role for the vitamin in energy metabolism. The active form of the vitamin, 1,25(OH)_2_D, acts through a nuclear vitamin D receptor (VDR). This receptor is expressed in many cells not directly involved in calcium metabolism [25]. VDR null mice demonstrate that the vitamin has a role in energy regulation, since these animals display a greater rate of energy expenditure, an increased β oxidation of fatty acids, up-regulation of uncoupling protein (UCP) and a leaner phenotype when compared to wild type mice [26,27]. In contrast to VDR null mice, however, the targeted expression of human VDR in adipocytes of transgenic mice resulted in a suppression of energy expenditure and fat oxidation leading to an obese phenotype [28]. The latter is also supported by a non-genomic action of the active metabolite, where 1,25(OH)_2_D stimulated enzymes controlling fat synthesis and reciprocally inhibited lipolysis in a human adipose tissue cell line [29]. The CYP27B1 gene is responsible for encoding the renal enzyme 1,α-hydroxylase, which in turn produces the active hormone from 25(OH)D. Mice lacking CYP27B1 also show lower body weights and reduced abdominal fat mass [25]. Based on the above one would conclude that a higher 1,25(OH)_2_D promoted adiposity [25,30].

However 1,25(OH)_2_D can regulate a spectrum of cellular processes, from proliferation, differentiation and development to apoptosis and secretion [31]. Sergeev [32] has documented that 1,25(OH)_2_D acted through intracellular increases in Ca^2+^ which in turn lead to increased apoptosis of adipocytes. As this was observed in mature adipocytes which are generally believed to be very stable, it has potential implications for human obesity. Follow up animal studies confirmed these findings, since supplementation of calcium and vitamin D reduced adiposity via increased adipocyte apoptosis [33]. Clearly this is a complex area and extrapolation from cellular and animal outcomes to the human condition is difficult. How does the rise in 25(OH)D from correcting vitamin D insufficiency in humans, affect obesity? Is its action through conversion to 1,25(OH)_2_D and hence downstream effects at the cellular level? Could it be acting through an improvement in calcium absorption? Or is the lowering of PTH central to these effects (Figure 1)? In a thought provoking paper, Heaney [3] has proposed that 25(OH)D has an endocrine action that subserves calcium metabolism, and also has a permissive autocrine role on intracellular 1,25(OH)_2_D. The key facet of the model is that without an optimal serum level of 25(OH)D the target cell is not enabled towards a proper functioning of its 1,25(OH)_2_D, and potential extra-skeletal effects [3]. If applied to human obesity this model would predict that supplementation with 1,25(OH)_2_D is unlikely to have an effect on adiposity, unless circulating 25(OH)D is at an optimal level. Deciding what is the optimal level of 25(OH)D for humans is then crucial. In reality, as is for bone health, there needs to be a close interplay between optimal calcium intake, optimal 25(OH)D, the suppression of PTH and action of 1,25(OH)_2_D [3].

There is a burgeoning literature on the potential role of vitamin D *per se* in the etio-pathogenesis of human obesity as well as other chronic diseases. Numerous cross-sectional studies have shown an inverse association between vitamin D and total body fat or visceral adiposity [34]. The predominant view is that being fat soluble, the vitamin is sequestered in the expanded adipose tissue mass and so results in an apparently lower level. Some confirmation comes from studies that report an improvement in vitamin status with weight loss [35,36], though the precise pathway for re-entry into the circulation needs investigation. There are very few RCTs that have examined the effect of vitamin D supplementation on a reduction in adiposity, so overall a consistent effect was not obtained [37]. More recently Vimaleswaran *et al*. [38] conducted a bi-directional Mendelian analysis in a very large number of individuals to ascertain causality between the vitamin D-body mass index (BMI) nexus. They concluded that obesity leads to a low vitamin D status but that any effect of a low status predisposing to a greater BMI was likely to be small. The corollary was also proven in a meta-analysis of studies on vitamin D supplementation and BMI [39]. Recently we questioned whether vitamin D supplementation had an effect on indices of body fatness in the absence of planned weight loss [40]. Our meta-analysis of high quality RCTs did not show any effect on percent fat or fat mass, though a marginal trend (*p* = 0.092) for BMI to decrease with increasing vitamin D status was noted. A potential decrease in BMI without a change in fat mass is intriguing when viewed in the absence of imposed caloric restriction. It could suggest a role for vitamin D in the mobilization of glycogen stores [40]. Overall, from the studies available to date, there is no consistent indication that improving vitamin D status caused a decrease in fat mass. Uncovering an effect of the vitamin on adiposity may require targeting a value of 25(OH)D well above current guidelines, and maintaining that value for a defined period.

## 4. Conclusions

The role of calcium and vitamin D in the regulation of energy balance has entered an exciting phase of research. The area receives inputs from epidemiology and clinical nutrition, as well as cellular and molecular research. A majority of the human evidence to date was designed with bone health in mind, and the optimal cut-off to determine vitamin D adequacy is still in discussion. It is our view that both calcium and vitamin D are functional nutrients in the battle against obesity. The drive to tilt the balance of evidence needs to continue, so expert committees acknowledge the extra skeletal effects of these nutrients.
